# Lactoferricin B Combined with Antibiotics Exhibits Leukemic Selectivity and Antimicrobial Activity

**DOI:** 10.3390/molecules29030678

**Published:** 2024-02-01

**Authors:** Jan Jakub Lica, Katarzyna Gucwa, Mateusz Heldt, Anna Stupak, Natalia Maciejewska, Natalia Ptaszyńska, Anna Łęgowska, Bhaskar Pradhan, Agata Gitlin-Domagalska, Dawid Dębowski, Joanna Jakóbkiewicz-Banecka, Krzysztof Rolka

**Affiliations:** 1 Department of Regenerative Medicine, Faculty of Medicine, Medical University of Warsaw, 02-091 Warsaw, Poland; 2 Department of Molecular Biochemistry, Faculty of Chemistry, University of Gdansk, 80-308 Gdansk, Poland; natalia.ptaszynska@ug.edu.pl (N.P.); anna.legowska@ug.edu.pl (A.Ł.); agata.domagalska@ug.edu.pl (A.G.-D.); dawid.debowski@ug.edu.pl (D.D.); krzysztof.rolka@ug.edu.pl (K.R.); 3 Department of Microbiology, Faculty of Biology, University of Gdansk, 80-308 Gdansk, Poland; katarzyna.gucwa@ug.edu.pl; 4 Department of Pharmaceutical Technology and Biochemistry, Faculty of Chemistry, Gdansk University of Technology, 80-233 Gdansk, Poland; mateusz.heldt@gmail.com (M.H.); nat.maciejewska@gmail.com (N.M.); 5 Polpharma Biologics S.A., Gdansk Science & Technology Park, 80-172 Gdansk, Poland; anna.stupak@polpharmabiologics.com; 6 Department of Biochemistry, Faculty of Pharmacy, Medical University of Warsaw, 02-097 Warsaw, Poland; bhaskar.pradhan@wum.edu.pl; 7 Department Medical Biology and Genetics, Faculty of Biology, University of Gdansk, 80-308 Gdansk, Poland

**Keywords:** alternative leukemia treatment, antileukemic agents, antimicrobial peptide, dual-activity compounds, bioconjugates, cell penetration peptide, ciprofloxacin, hematopoietic stem cell transplantation, levofloxacin, lactoferricin B, fluconazole, fluoroquinolones

## Abstract

The fusion of penetrating peptides (PPs), e.g., cell penetration peptides (CPPs) or antimicrobial peptides (AMPs), together with antimicrobial agents is an expanding research field. Specific AMPs, such as lactoferricin B (LfcinB), have demonstrated strong antibacterial, antifungal, and antiparasitic activity, as well as valuable anticancer activity, proving beneficial in the development of anticancer conjugates. The resulting conjugates offer potential dual functionality, acting as both an anticancer and an antimicrobial agent. This is especially necessary in cancer treatment, where microbial infections pose a critical risk. Leukemic cells frequently exhibit altered outer lipid membranes compared to healthy cells, making them more sensitive to compounds that interfere with their membrane. In this study, we revisited and reanalyzed our earlier research on LfcinB and its conjugates. Furthermore, we carried out new experiments with a specific focus on cell proliferation, changes in membrane asymmetric phosphatidylserine location, intracellular reactive oxygen species (ROS) generation, mitochondrial functions, and in vitro bacterial topoisomerase inhibition.

## 1. Introduction

The possibility of combining different types of chemical compounds has given rise to the development of new molecules with improved pharmacological characteristics. Among these are peptides and antimicrobial conjugates, which offer expanded molecular targets, reduced toxicity, and the ability to overcome drug resistance when compared to their original substances [1,2,3,4,5,6,7,8]. CPPs and AMPs are two types of PPs that demonstrate not only antimicrobial properties but also anticancer potential [2,4,9,10,11,12,13,14,15,16,17]. Table 1 showcases the functional classification of PPs, and Table 2 outlines the methodology for classification.

The impact of PPs on biological systems can be altered, improved, and prolonged by making structural modifications to the peptide itself or by linking it to other compounds. As previously demonstrated, CPPs such as TP10 and its fluoroquinolone conjugates show antibacterial and antileukemic effects, minimal generation of ROS, and a slight impact on mitochondrial functions [8,16]. Crucially, leukemic cells frequently display disturbances in the outer lipid membrane, making them highly sensitive to compounds that interfere with membrane integrity [18,19,20,21,22,23,24,25]. These lipid disturbances have been identified as a cellular target for hematological malignancies [26,27,28,29,30]. Microbial infections are a significant contributing factor to the morbidity and mortality of acute leukemia (AL) patients, with a median incidence reaching around 27% [31]. This issue is particularly pronounced in immune-deficient patients, such as children with AL, and older patients with various types of leukemia [32,33,34,35,36]. For this reason, treatment requires the use of immunosuppressive drugs, which in turn increases the risk of microbial infections. The standard antimicrobials employed in this context are ciprofloxacin (CIP) and levofloxacin (LVX) [37,38,39]. It is well established that the ability of CIP and LVX to exhibit antibacterial activity relies on the inhibition of topoisomerase (a mechanism also applied in cancer treatment) [40,41,42]. Unfortunately, both CIP and LVX demonstrate some unwanted effects, including toxicity induced by ROS [43,44,45,46].

Our data, obtained from fluoroquinolones conjugated to TP10 [8,16], as well as chimera peptides composed of human neutrophil peptide (HNP) [43], demonstrate selectivity against both microbes and leukemic cells, as opposed to healthy human cell cultures. To investigate the anticancer efficacy of a PP other than CPPs (TP10 and NHP) combined with antibiotics, we selected lactoffericin B (LfcinB), an AMP with established anticancer activity and low toxicity [47,48,49,50,51,52,53,54]. LfcinB is a shorter version of lactoferrin, and like the original peptide, it demonstrates anticancer properties [55,56,57,58,59,60,61,62].

In this latest update, delving deeper into the previously discussed results regarding the biological effects of LfcinB and its combination with an antibiotic [48], we conducted additional MTT tests specifically for compound **4** (LfcinB covalently linked to fluconazole (FLC)), focusing on the HL60 and HEK293 cell lines. Furthermore, we calculated the Bacteriostatic Selectivity Index (B_SI_), Fungistatic Selectivity Index (F_SI_), and Malignancy Selectivity Index (M_SI_). To assess the basal molecular impact of LfcinB and selected conjugates on bacteria, we examined *S. aureus* alterations in the membrane asymmetric distribution of phosphatidylserine. Additionally, we conducted in vitro bacterial topoisomerase inhibition assays. Furthermore, we expanded our research on potential toxicity by examining intracellular ROS generation in HL60 and HEK293 and assessing their influence on mitochondrial functions.

Highlighting their leukemic selectivity and antimicrobial activity, along with a minimal impact on ROS generation and mitochondrial function, we have identified an interesting compound, designated as number 3 (disulfide bridge between LfcinB and CIP). This conjugate demonstrates all these properties at an IC_50_ in micromolar concentrations and an EC_50_ (effective concentration; IC normalized to cell number [63]) in the nanomolar range. It is worth noting that conjugate 3 serves as an example of a disulfide connection between parenteral drugs, representing a promising target for enzymatic reduction.

## 2. Results

### 2.1. Inhibition of Cancer Cell Proliferation and EC_50_ of Lfcin and Its Conjugates

To enhance the previously published dataset on the inhibition of cancer cell growth by LfcinB and its conjugates [48], we conducted additional experiments for conjugate 4 on HL60 and HEK293. The antiproliferative effects of LfcinB and its conjugate 4 were evaluated using the MTT assay, and the calculated IC_50_ values are detailed in Supplementary Table S1. However, the cellular response to compound **4** for HL60 was approximately 65 μM. It is noteworthy that the IC_50_ of the leukemic cells was still approximately 2.5 times lower than that observed in healthy HEK293 cells.

**Figure 1:**
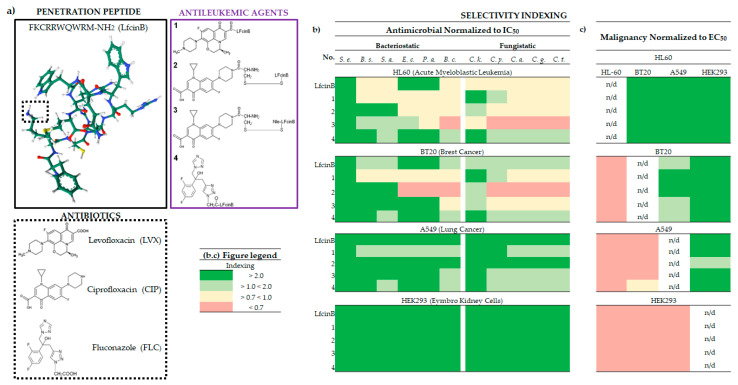
Compound structures and selectivity indexing panels. (**a**) Chemical structures of LfcinB, antibiotics, and conjugates; LfcinB structure was generated by https://www.rcsb.org/ (accessed on 30 November 2023); Peptide SMILES was generated by https://www.novoprolabs.com/ (accessed on 30 November 2023); (**b**) Antimicrobial selectivity normalized to IC_50_ HL60; (**c**) Malignancy selectivity normalized to EC_50_; Selectivity indexing: Red—none, Yellow—moderate, Light green—good, Deep green—very good; IC_50_—Inhibitory concentration; EC_50_—Effective concentration (IC normalized to cell number); Abbreviations: S. e.—Staphylococcus epidermidis ATCC 12228; B. s.—Bacillus subtilis ATCC 6633; S. a.—Staphylococcus aureus ATCC 25923; Escherichia coli ATCC 25922; P. a.—Pseudomonas aeruginosa ATCC 27853; B. c.—Bacillus cereus PCM 2003; C. k.—Candida krusei DSM 6128; C. p.—Candida parapsilosis DSM 5784; C. a.—Candida albicans ATCC 10231; C. g.—Candida glabrata DSM 11226; C. t.—Candida tropicalis CZD 519. For more detailed information please refer to Supplementary Table S2.

When it comes to calculating and comparing EC_50_ values for LfcinB and all its conjugates (Figure 1a) on HL60, BT20, A549, and HEK293 cell lines, conjugates 1 (covalently bonded LfcinB and LVX) and 3 proved to be the most active against cancer cells, with EC_50_ values ranging from 3 to 69 nM (Supplementary Table S2). By contrast, conjugate 4 exhibited an EC_50_ of 108 nM in healthy HEK293 cells. Notably, the EC_50_ values for the FLC conjugate were approximately twice as high as those for conjugates containing CIP and LVX on all tested cancer cell lines (Supplementary Table S2). The most significant growth inhibition was observed for conjugate 3 on HL60, with an EC_50_ of 3 nM, while the highest values were recorded for LfcinB (499 nM) and conjugate 3 (409 nM) in HEK293. The most sensitive cell line to the action of LfcinB and all tested conjugates was HL60, with EC_50_ values ranging from 3 to 13 nM; the least sensitive turned out to be HEK293, with EC_50_ values ranging from 108 to 499 nM (Supplementary Table S2).

### 2.2. The Biological Selectivity of LfcinB and Its Conjugates

#### 2.2.1. Bacteriostatic Selectivity

B_SI_ values were analyzed for the following bacteria strains: *S. epidermidis*, *B. subtilis*, *S. aureus*, *E. coli*, *P. aeruginosa*, and *B. cereus*, as well as the following cell cultures: leukemia (HL60), breast cancer (BT20), non-small-cell lung cancer (A549), and non-cancerous embryonic kidney cells (HEK293), represented by MIC/IC_50_ (Supplementary Table S2) and B_SI_ indexing as illustrated in Figure 1b.

LfcinB and conjugate 3 demonstrated the most favorable B_SI_ values across all tested cell lines and bacterial strains (Figure 1b). *S. epidermidis* exhibited highly favorable B_SI_ values of >5 and <472, while *B. cereus* showed the least favorable B_SI_ values of <0.7 and <16 (Figure 1b and Supplementary Table S2). For HEK293, all B_SI_ calculations for LfcinB and its conjugates indicated high favorability (>2) against all tested strains. Conjugate 3 achieved the best score against *S. epidermidis* with a B_SI_ > 471, and LfcinB showed a B_SI_ > 179.

The least favorable index was noted for the HL60 cell line, where, apart from the values for S. epidermidis, with B_SI_ values ranging from >6 to <13, the index varied from unfavorable (B_SI_ > 0.7), as seen in the case of conjugate 3 on *B. cereus*, to very good (B_SI_ > 13 and <14), as observed with conjugate 2 (alternative disulfide bridge connection between peptide and CIP) on *S. epidermidis*. Mostly the index was not more than around 3, mainly remaining approximately around 0.7 (Figure 1b and Supplementary Table S2). This outcome shows that, in HL60, the bacteriostatic effect is less efficient than the cytostatic effect obtained on leukemic cells. For the BT20 cell line, the B_SI_ results were similar to those for HL60, with the cancer cytostatic effect, especially for conjugates 1 and 2, surpassing the bacteriostatic one (Figure 1b). Notably, the difference in the action of conjugates 2 and particularly 3 (connected by disulfide bonds) between HL60 and BT20 for *E. coli*, *P. aeruginosa*, and *B. cereus* suggests that such a difference in combination might be associated with a more selective cytostatic effect against leukemia (Figure 1b).

#### 2.2.2. Fungistatic Selectivity

F_SI_ values were analyzed for the following bacteria strains: *C. krusei*, *C. parapsilosis*, *C. albicans*, *C. glabrata*, and *C. tropicalis*, as well as the following cell cultures: HL60 (leukemia), BT20 (breast cancer BT20), non-small-cell lung cancer (A549), and HEK293 (non-cancerous embryonic kidney cells), represented by MIC/IC_50_ (Supplementary Table S2) and F_SI_ indexing in Figure 1b.

Conjugate 4 (containing FLC) and LfcinB exhibited the most desirable F_SI_ values across all tested cell lines and bacterial strains (Figure 1b). *C. krusei* showed favorable F_SI_ values of >7 and <34, while the other three strains demonstrated lower F_SI_ values of <0.7 and <18 (Figure 1b and Supplementary Table S2). Similarly, for HEK293, all F_SI_ calculations for LfcinB and its conjugates indicated favorability (>2) against all tested strains. Conjugate 3 exhibited a favorable F_SI_ ranging from >15 to <31 for all strains.

The least favorable index was noted for HL60 (Figure 1b). The index was mostly unfavorable (F_SI_ > 0.7) or moderate (<0.7 and >1), and very good in the case of *C. krusei* for compounds **1** and **4**, with F_SI_ values of 2.9 and 2.4, respectively. As for B_SI_, the fungistatic effect was lower than the antiproliferative effect on leukemic culture.

The F_SI_ results for BT20 resembled those for HL60. The main differences observed across all strains were a more favorable F_SI_ for BT20 with LfcinB and conjugate 3, while it was less favorable for conjugate 2. Therefore, as in the case of B_SI_, the differences in F_SI_ may suggest a more selective cytostatic effect against leukemic cells (Figure 1b).

#### 2.2.3. Malignancy Selectivity

To evaluate their effect on leukemia, cancer, and non-cancer cells, the Malignancy Selectivity Index was calculated as M_SI_ = EC_50_ (non-cancerous)/EC_50_ (leukemic or cancerous) or EC_50_ (leukemic or cancerous)/EC_50_ (leukemic or cancerous). These data are presented in Supplementary Table S3 and visualized in Figure 1c. Most compounds displayed higher M_SI_ values for cancerous, especially leukemic, cells than for healthy cells (Figure 1c, Supplementary Table S3).

M_SI_ values for all compounds reached their peak for HL60, ranging between >3 and the highest score of <136, which was obtained for compound **3** on HEK293 (refer to Supplementary Table S3). LfcinB also displayed promising M_SI_ parameters for HL60, attaining values of between >8 and <52. Furthermore, disulfide bridge conjugates 2 and 3 exhibited higher M_SI_ values in HL60 than was the case for their counterpart 1 and without the bridge connection (see Figure 1c). HL60 was the only cell line that exhibited favorable M_SI_ values across all compounds when compared to the two other cancer cell lines and the non-cancerous HEK293 cells. Notably, all the compounds demonstrated selectivity towards healthy cells, indicated by negative M_SI_ values (Figure 1c).

### 2.3. LfcinB and Its Conjugates Eliminating Bacteria

#### 2.3.1. Switch the Asymmetry in Membrane Phospholipids

**Figure 2:**
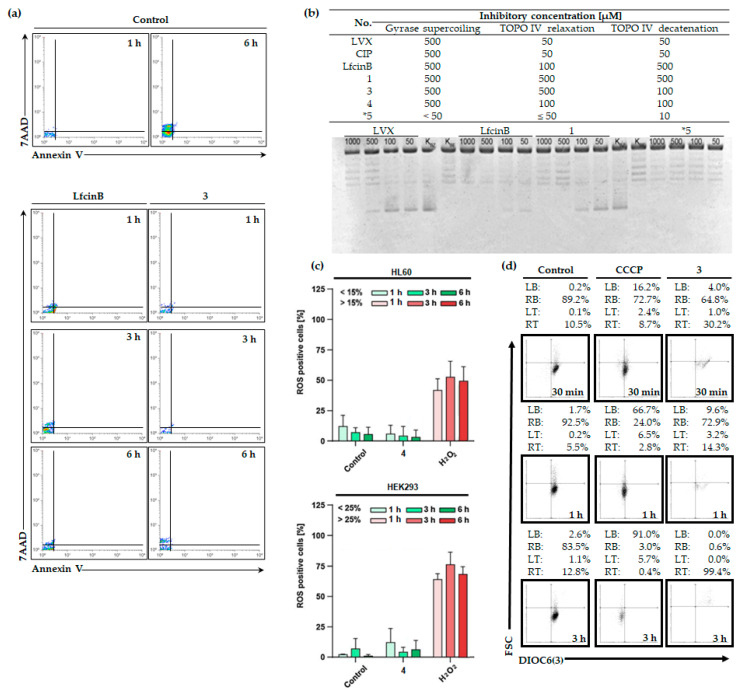
Effectiveness of LfcinB and its bioconjugates in biological systems. (**a**) The bactericidal activity of LfcinB and its bioconjugate 3. Two-parameter dot plots showcase the shift in the asymmetric localization of phosphatidylserine in the external membrane induced by the compound in *S. aureus* (Annexin V-positive events derived from the right top and bottom squares of the discriminator). The outcomes of the flow cytometry were analyzed with the free online software Floreada.io (https://floreada.io/ (accessed on 30 November 2023; the last update was carried out in June 2023)). Representative data are based on three independent repetitions. (**b**) The compound hinders the supercoiling activity of gyrase. Controls include K_BZ_, representing the sample without the compound, and K_BE_, representing the sample without the enzyme. The compounds underwent testing within the range of 1000 to 50 μM. For more data on the additional compound *5, please refer to [43]. (**c**) The impact of LfcinB and its conjugate 4 on intracellular ROS generation in HL60 and HEK293 measured using CM-H2DCFDA. (**d**) Dot plots illustrating the mitochondrial potential of HL60-treatedcompound 3, assessed through DIOC6(3) staining. A positive control with 50 μM CCCP was applied. LB, left bottom; RB, right bottom; LT, left top; RT, right top. Flow data were analyzed by Flowing Software 2.5.1 (Turku Bioscience, Turku, Finland). Values represented from three independent experiments.

Our study incorporates insights from Dwyer’s research, emphasizing that bacteria can display essential characteristics of eukaryotic cell death [64]. In the dot plots shown in Figure 2a (7AAD vs. Annexin V), there is a notable reduction in the number of events over 6 h in samples treated with compounds compared to the untreated control. Specifically, when compared to LfcinB, the impact of conjugate 3 (at MIC concentrations) is more pronounced, as reflected in a lower number of events from the first hour to 6 h. At this point in time, we observed essential disturbances in the asymmetric distribution of phosphatidylserine in the membrane (as indicated by the Annexin V level), leading to a loss of integrity (evidenced by 7AAD accumulation) and subsequent cell death. Consequently, the bacteriostatic effect induced by the compound also manifests as bactericidal (Figure 1b and Figure 2a).

#### 2.3.2. DNA Gyrase and Topoisomerase IV Inhibition

Disruptions in the membrane, triggered by the treatment with compounds, result in a loss of cell integrity, enhancing the entry of these compounds into the cell. Our study indicates that topoisomerase IV serves as the primary target for quinolones and their conjugates. We evaluated seven compounds for their inhibitory effects on *S. aureus* topoisomerases using three assays: gyrase activity, relaxation, and topoisomerase IV activity (Figure 2b). The LfcinB peptide alone exerted the most significant impact on topoisomerase IV relaxation activity, while compound *5 (chimera CH9 of HNP-1; for more details please refer to [43]) demonstrated the highest inhibitory effect among tested compounds (Figure 2b). Notably, LfcinB, when conjugated with CIP, exhibited a relatively high inhibitory effect on all enzyme activities, compared to its connection to LVX (Figure 2b).

### 2.4. LfcinB and Its Conjugates Exhibit Low Toxicity in Human Cell Lines

To verify whether the observed leukemic-selective cytostatic effect is potentially toxic, we expanded our previous studies [48] to include the evaluation of intracellular ROS generation by compound **4** (Figure 2c). Furthermore, we investigated the impact of the selected conjugate on mitochondrial functions (Figure 2d). The ROS-generating activity of conjugate 4 with FLC was evaluated in the HL60 and HEK293 cell lines (Figure 2c). We employed the fluorescent CM-H2DCFDA probe for ROS detection, with H_2_O_2_ used as a positive control due to its known role as an ROS inducer. Incubation with compound **4** (at 2-fold IC_90_) resulted in negligible levels of cellular ROS (Figure 2c). Importantly, these findings align with the data reported for conjugates 1–3, as detailed in our earlier publication [48]. The obtained evidence strongly suggests that the cytostatic effect is ROS-independent (Figure 2c). To investigate further, we examined whether the compounds could cause mitochondrial dysfunction. To confirm this, we assessed mitochondrial potential through flow cytometry using HL60 cells stained with DIOC6(3) and treated with compound **3** (Figure 2d). Our analysis showed that the tested conjugate had a minimal impact on mitochondrial potential, suggesting a low risk of toxicity in this context.

## 3. Discussion

### 3.1. Antimicrobial Effect

AMPs are small, positively charged molecules with amphipathic structures. They are found widely in animal tissues and serve as a crucial first line of defense against infections. AMPs exhibit a broad spectrum of activity, mainly targeting bacteria, fungi, and viruses [65], and this phenome extends to LfcinB and its conjugates as well (Figure 1a,b). The primary mode of action involves the direct disruption of microbial cell membranes and selective binding to negatively charged phospholipid head groups on bacterial membranes [66]. According to our findings, this type of effect occurs not only for LfcinB (Figure 2a). Importantly, AMPs are not attracted to neutral host cell membranes [67]. This effect might have influenced the low activity of all tested compounds against non-cancerous HEK293 cells and their significantly higher activity against cancerous and leukemic cells (Figure 1c).

In addition to membrane disruption, AMPs can impact microbial cells through various mechanisms, including interactions with intracellular targets such as nucleic acids or proteins, as well as the disruption of cellular processes. Fluoroquinolones exert their antimicrobial effect by inhibiting two essential enzymes involved in bacterial DNA synthesis: DNA gyrase and topoisomerase IV [68]. DNA gyrase introduces negative supercoils in bacterial DNA, facilitating the separation of the daughter chromosomes necessary for replication initiation. Topoisomerase IV is responsible for the decatenation process, separating daughter chromosomes at the end of replication [69,70]. Fluoroquinolones form complexes with either DNA gyrase or topoisomerase IV bound to bacterial DNA, causing conformational changes that deactivate these enzymes. This interference impedes the progression of the replication fork and bacterial DNA synthesis, ultimately leading to rapid bacterial cell death [68]. Figure 2b illustrates the inhibition of the DNA-gyrase in Gram-negative and DNA-topoisomerase IV in Gram-positive bacteria in an in vitro assay induced by fluoroquinolones.

The impact of lactoferrin on gut microbiota and toll-like receptors (TLRs) in mice undergoing microbiota imbalance due to antibiotic treatment was also published [71]. The effects of antibiotic therapy on gut microbiota were explored, emphasizing the potential for dysbiosis, a disruption in microbial balance. The primary focus was on understanding how the composition of gut microbiota and TLR expression was regulated by lactoferrin. Concluding insights underscored the significance of the findings, emphasizing the intricate interplay between lactoferrin, gut microbiota, and the immune system.

### 3.2. Antileukemic Selectivity and Low Potential Toxicity

Leukemic cells, sensitive to compounds disrupting the outer lipid membrane, represent a target for hematological malignancies [72]. As we previously demonstrated with CPP, TP10, and its conjugates with CIP and LVX, these compounds exhibit selective and low toxicity toward leukemic cells [16]. Consequently, certain AMPs may also be active against these cell types. Our research revealed that LfcinB and its conjugates exhibit selectivity towards leukemic cells, antimicrobial activity, and a minimal impact on ROS generation and mitochondrial function (Figure 1 and Figure 2c,d). These AMPs, when combined with antimicrobial agents, demonstrate potential for effectively targeting leukemic cells and managing infections during HCT.

In terms of structure, the distinction between conjugates and a mixture of LfcinB with an antibiotic (CIP or LVX or FLC) is characterized by the presence of a covalent or disulfide bridge bond linking LfcinB to the antibacterial or antifungal agent. In the case of conjugates, delivery inside cells is coordinated and potentially improved, as the cell membrane can be made permeable by the presence of conjugate oligomers. These conjugates, located within cellular components, serve as effective targets for enzymatic reduction, leading to the subsequent release of individual components within the cell, binding to certain targets and causing damage to cellular pathways. Various mechanisms may contribute to making conjugates more advantageous in vivo, aspects that prove challenging to discern within an in vitro environment. These factors encompass co-targeting specific tissues, heightened metabolic stability, decreased toxicity prior to linker cleavage, and a diminished likelihood of developing resistance during prolonged treatment. It is important to note that these considerations remain speculative until promising candidate drugs are selected for animal studies.

Analyzing data from lactoferrin studies in both rats [73] and humans [74,75,76,77], as well as talactoferrin [78] studies in humans, indicates that LfcinB can be classified as biologically safe. There were no reported hematological, hepatic, or renal toxicities during therapy. Notably, 88% of patients observed a reduction in their tumor growth rate. The research also revealed a positive correlation with the immune system through binding mechanisms [79,80]. LfcinB demonstrates potential immunomodulatory functions, influencing the host’s immune response. By enhancing the immune system’s ability to recognize and eliminate pathogens, LfcinB contributes to overall biological safety by fortifying the body’s natural defense mechanisms. The peptide’s stability and resistance to enzymatic degradation further contribute to its biological safety. These characteristics ensure the sustained availability of LfcinB at the site of action, optimizing its therapeutic efficacy while minimizing potential systemic side effects.

## 4. Materials and Methods

### 4.1. Drugs

The following compounds were purchased from Sigma-Aldrich (St. Louis, MO, USA): 7-aminoactinomycin (Cat. A9400), CCCP (Cat. 215911), ciprofloxacin (Cat. 17850), levofloxacin (Cat. 1362103), DMSO (Cat. D8418), and LfcinB. Conjugates 1–4 were synthesized using the method detailed in our previous work [48]. The characterizations of these conjugates, including HPLC purities and mass spectroscopy, are presented in Supplementary Table S5 and Supplementary Figure S1. Compound *5 was synthesized according to the procedure described in Ptaszyńska et al. (2018). All drugs were dissolved in DMSO at concentrations ranging from 2 to 10 mM and stored at −20 °C. The 7-AAD was dissolved in MeOH:H_2_O (4:6) and stored at 4 °C.

### 4.2. Cell Cultures

HL60, CEM, and A-549 were acquired from ATCC. HL60 was cultured in RPMI-1640 medium (Cat. R5158, Merck, Rahway, NJ, USA) and HEK293 Eagle’s Minimum Essential Medium (Cat. M5775, Merck). Both lines were supplemented with 10% FBS (Cat. F7524, Sigma-Aldrich), 2 mM L-glutamine (Cat. G8540, Sigma-Aldrich), and the antibiotics penicillin (100 U/mL) and streptomycin (100 mg/mL) at 37 °C in a humidified atmosphere of 5% CO_2_ and 95% air. Cell lines were routinely screened for mycoplasma contamination. Cell density was measured using a Coulter Z2 (Beckman, Brea, CA, USA) equipped with a 100 µm aperture or using the flow cytometer Guava EasyCyte 8HT (Merck-Millipore, Burlington, MA, USA).

### 4.3. Determination of Drug Cytotoxicity

As mentioned in [61], we followed detailed procedures, including the composition of media and growth conditions, for the application of conjugate 4 on both HL60 and HEK293 in the MTT drug sensitivity assay. The initial cellular density for cultures seeded in a 96-well plate was 5000 cells/well for HL60 and 1500 cells/well for HEK293.

### 4.4. Switch of S. aureus Asymmetric Phosphatidylserine Location

Bacterial cultures with an OD200, as utilized in bacteriostatic experiments (Ptaszyńska et al., 2019 [16,48]), were plated on Petri dishes. Compounds were added at the MIC concentration to the test sample, while no compound was added to the negative control. After an appropriate incubation period (45 min, 2.45 min, and 5.45 min), samples were collected and promptly washed with PBS. The pellet was then suspended in 100 μL of annexin binding buffer (BioSource International, Camarillo, CA, USA), which included 0.25 μL of 7-AAD (7-aminoactinomycin D, 1 mg/mL, Sigma) and 1 μL of Annexin–FITC (Thermo Fisher, Waltham, MA, USA). Following the addition of 400 μL of annexin binding buffer, the samples were left in the dark for 15 min. Subsequently, the samples were placed on ice and immediately read by the Guava EasyCyte (Merck-Millipore, Burlington, MA, USA). Flow results were analyzed with the free online software https://floreada.io/ (accessed on 30 November 2023; the last update was carried out in June 2023).

### 4.5. Inhibition of S. aureus Gyrase and Topoisomerase IV Activities

Inhibitory concentrations for three gyrase and topoisomerase IV activities were assessed: gyrase supercoiling, TOPO IV relaxation, and TOPO IV decatenation. For this, commercially available kits were used (*S. aureus* gyrase supercoiling assay kit, *S. aureus* topoisomerase IV relaxation assay kit, and topoisomerase IV decatenation kit all purchased from Inspiralis (Norwich, UK)). The procedure was carried out according to the manufacturer’s instructions. The substrates for the reactions were 500 ng of the relaxed pBR322 plasmid, 500 ng of supercoiled pBR322, or 200 ng of kinetoplast DNA (kDNA) from Crithidia fasciculate, respectively. The studied compounds were dissolved and diluted in dd H_2_O at the experimentally selected concentrations (gyrase: 2000–250 μM for CIP, 1000–50 μM—LVX, 500–50 μM conjugates; topoisomerase: 500–5 μM CIP/LVX, 250–10 μM—conjugates). Appropriate controls were included (with or without the enzyme or without the addition of the tested compound). After the addition of the indicated amount of the enzyme, the reaction proceeded at 37 °C for 30 min. Loading buffer was used for reaction termination. The studied compounds were extracted from the reaction mixtures with 30 µL of a chloroform/isoamyl alcohol solution (24:1; *v*/*v*). After centrifugation (3 min, 20,000× *g*), the upper aqueous phase was loaded onto 1% agarose gels and run at 90 V for 4 h in TBE buffer (90 mM Tris-base, 70 mM boric acid, 1 mM EDTA, pH 8). Gels were stained with 1 μg/mL ethidium bromide (Sigma, Burbank, CA, USA) for 15 min to visualize the DNA, and unbound ethidium bromide was removed by washing the gel in dd H_2_O for 15 min.

### 4.6. Intracellular ROS Generation

Cell lines were cultured as mentioned above and seeded in Petri dishes (Falcon, Cary, NC, USA) in the amount of 25 × 10^3^ per Φ35 dish. HEK293 cells were allowed to attach overnight. ROS generation potential was tested for selected compounds after 0.25, 0.5, 1, 3, 6, and 24 h of incubation. The CM-H2DCFDA molecular probe (Thermo Fisher Scientific, Waltham, MA, USA) at a final concentration of 1 µM was added 15 min before the analysis. After staining, HEK293 cells were trypsinized, harvested, and suspended in fresh media. The 7AAD (Cat. A9400, Sigma Aldrich) was added just before analysis. Analyses were carried out with a Guava EasyCyte flow cytometer (Merck-Millipore, USA). Flow cytometry data were processed with Flowing Software 2.5.1 (Turku Bioscience, Turku, Finland).

### 4.7. Investigation of Mitochondrial Potential

HL60 was incubated with 100 nM 3,3′-dihexyloxacarbocyanine iodide (DiOC6(3)) (Cat. D273, Thermo Fisher Scientific). Cells were stained under culture conditions for 15 min, washed with prewarmed PBS, and examined by Guava EasyCyte flow cytometer (Merck-Millipore, USA). The results of flow cytometry were analyzed with Flowing Software 2.5.1 (Turku Bioscience, Finland).

## 5. Conclusions

Combining PPs with antimicrobial agents shows potential for effectively targeting leukemic cells and addressing infections during HCT. So far, there have not been similar applications of such compounds in leukemia treatment. The conventional approach, as seen in AML, still relies on the use of non-specific therapies while simultaneously aiming for autologous transplantation. In the processes of conditioning, myeloablation, and immunosuppression, cytostatics such as cyclophosphamide [81,82,83,84,85,86,87] and preventive antibiotics such as CIP or LVX are employed. Cyclophosphamide exhibits low selectivity and is combined with the administration of antibiotics, resulting in a less intense synergistic effect and less tissue-specific availability compared to LfcinB. We believe that our research will involve the generation of new structures, which can successfully be applied in the development of therapy for treating leukemia and infections. The optimization of these structures can occur through the careful selection of the PP type and the attached bacterial or anticancer compound.

## Figures and Tables

**Table 1:** Functional classification of penetration peptides (PPs).

Penetration Peptides Classification			
**AMP Type**	**Biological Effect**		
	**Anticancer**	**Antimicrobial**	**Healthy Cells**
Canonical	Poor	Significant	Poor
Transporters	Poor	Poor	Poor
Dual Activity	Significant	Significant	Poor
Toxic	Poor	Poor	Significant
**CPP Type**	**Biological Effect**		
	**Anticancer**	**Antimicrobial**	**Healthy Cells**
Canonical	Poor	Poor	Poor
Antimicrobial	Poor	Significant	Poor
Anticancer	Significant	Poor	Poor
Dual Activity	Significant	Significant	Poor
Toxic	Poor	Poor	Significant

**Table 2:** Methodology for classification of penetration peptides (PPs). *E/NEM—epithelial/non-epithelial malignancy; EC—effective concentration; IC—inhibitory concentration; FIC—fungistatic inhibitory concentration; *NEM—non-epithelial malignancy; MIC—minimal inhibitory concentration.

No.	Biological Effects of Penetration Peptides Predicted by Selectivity Index			
**1**	Conduct the Cell Proliferation Tests for Determining Pharmacological Values			
	Human Cell Lines		Bacteria	Fungi
	IC_50_	EC_50_	MIC	FIC
**2**	Antimicrobial Effect Determined by Selectivity Index			
	Bacteriostatic (B_SI_)		Fungistatic (F_SI_)	
	MIC/IC_50_ < 0.7 Poor		FIC/IC_50_ < 0.7 Poor	
	MIC/IC_50_ > 0.7 < 1.0 Moderate		FIC/IC_50_ > 0.7 < 1.0 Moderate	
	MIC/IC_50_ > 1.0 < 2.0 Good		FIC/IC_50_ > 1.0 < 2.0 Good	
	MIC/IC_50_ > 2.0 Significant		FIC/IC_50_ > 2.0 Significant	
**3**	Antimalignancy Effect Determined by Selectivity Index			
**3a**	Anticancer			
	EC50 Cancer/EC50 of Healthy or *NEM Cells < 0.7 Poor			
	EC50 Cancer/EC50 of Healthy or *NEM Cells > 0.7 < 1.0 Moderate			
	EC50 Cancer/EC50 of Healthy or *NEM Cells > 1.0 < 2.0 Good			
	EC50 Cancer/EC50 of Healthy or *NEM Cells > 2.0 Significant			
**3b**	Antileukemic			
	EC_50_ Leukemia/EC_50_ of Healthy or *E/NEM Cells < 0.7 Poor			
	EC_50_ Leukemia/EC_50_ of Healthy or *E/NEM Cells > 0.7 < 1.0 Moderate			
	EC_50_ Leukemia/EC_50_ of Healthy or *E/NEM Cells > 1.0 < 2.0 Good			
	EC_50_ Leukemia/EC_50_ of Healthy or *E/NEM Cells < 2.0 Significant			
**4**	Neutral Transporters			
	EC_50_ Healthy/EC_50_ of *E/NEM Cells < 0.7 Poor			
	EC_50_ Healthy/EC_50_ of *E/NEM Cells > 0.7 < 1.0 Moderate			
	EC_50_ Healthy/EC_50_ of *E/NEM Cells > 1.0 < 2.0 Good			
	EC_50_ Healthy/EC_50_ of *E/NEM Cells < 2.0 Significant			

## Data Availability

Data are contained within the article and Supplementary Materials.

## Supplementary Materials

The following supporting information can be downloaded at: https://www.mdpi.com/article/10.3390/molecules29030678/s1. Supplementary Table S1. Cellular effect of compound no. 4; Supplementary Table S2. Effective concentration values.; Supplementary Table S3. Antimicrobial Selectivity Index of LfcinB and selected conjugates in relation to IC_50_ values for HL60, BT20, A549, and HEK293; Supplementary Table S4. Normalized to EC_50_ Malignancy Selectivity Index; Supplementary Table S5. Physicochemical properties of peptide conjugates and its components; Supplementary Figure S1. MS analysis of peptide and peptide conjugates and their constituents.
