# Supplementary material for: Lactoferricin B Combined with Antibiotics Exhibits Leukemic Selectivity and Antimicrobial Activity
Source: Molecules. 2024 Feb 1;29(3):678. doi: 10.3390/molecules29030678 (PMC10856415; doi:10.3390/molecules29030678)
Supplement: Supplementary file 1 [file molecules-29-00678-s001.zip › molecules-2788882-supplementary.pdf]

molecules

MDPI

Supplementary Materials

Inhibition of Cellular Proliferation by No. 4 IC_{50} [µM]	
HL60	HEK293
64.9	161.3

Supplementary Table S1. Cellular effect of compound no. 4. MTT test demonstrating the ant-leukemic effects of Conjugate 4 compared to its impact on non-cancerous embryo cells after 72 hours of incubation; The initial cellular density for HL60 was 5,000 cells, and for HEK293, it was 1,500 cells per well, respectively; IC_{50} - Inhibitory Concentration; Values represent averages ±SEM from five independent biological experiments in technical triplicate; n/d - not determined.

Efective Concentration IC_{50} [nM]				
No.	HL60	BT20	A549	HEK293
LfcinB	10	77	151	499
1	8	24	69	307
2	8	22	257	322
3	3	22	37	409
4	13	40	50	108

Supplementary Table S2. Effective concentration values. The conversion from IC_{50} to EC_{50} was performed following the method outlined in [62]; For IC_{50} values, please refer to Supplementary Table ST1 and the corresponding reference [61]; IC_{50} - Inhibitory Concentration; EC_{50} - Effective Concentration.

Antimicriobial Selectivity Index											
No.	Bacteriostatic Normalized to IC_{50}						Fungistatic Normalized to IC_{50}				
	HL60										
	S. e.	B. s.	S. a.	E. c.	P. a.	B. c.	C. k.	C. p.	C. a.	C. g.	C. t.
LfcinB	11.5	0.7	0.7	2.9	2.9	0.7	0.7	0.7	0.7	0.7	0.7
1	5.7	0.7	0.7	0.7	0.7	0.7	2.9	1.4	0.7	0.7	0.7
2	12.9	3.2	3.2	0.8	0.8	0.8	1.6	0.8	0.8	0.8	0.8
3	11.6	1.5	1.5	1.5	0.7	0.4	0.7	0.4	0.4	0.4	0.4
4	9.5	2.4	1.2	4.8	4.8	1.2	2.4	1.2	1.2	1.2	1.2
	BT20										
LfcinB	27.5	1.7	1.7	6.9	6.9	1.7	1.7	1.7	1.7	1.7	1.7
1	5.2	0.7	0.7	0.7	0.7	0.7	2.6	1.3	0.7	0.7	0.7
2	10.3	2.5	2.5	0.6	0.6	0.6	1.3	0.6	0.6	0.6	0.6
3	25.1	3.2	3.2	3.2	1.6	0.8	1.6	0.8	0.8	0.8	0.8
4	8.8	2.2	1.1	4.4	4.4	1.1	2.2	1.1	1.1	1.1	1.1
	A549										
LfcinB	53.9	3.4	3.4	13.6	13.6	3.4	3.4	3.4	3.4	3.4	3.4
1	15.3	1.9	1.9	1.9	1.9	1.9	7.7	3.8	1.9	1.9	1.9
2	120.6	29.7	29.7	7.5	7.5	7.5	14.9	7.5	7.5	7.5	7.5
3	43.2	5.5	5.5	5.5	2.8	1.4	2.8	1.4	1.4	1.4	1.4
4	11.0	2.8	1.4	5.5	5.5	1.4	2.8	1.4	1.4	1.4	1.4
	HEK293										
LfcinB	178.1	11.2	11.2	44.8	44.8	11.2	11.2	11.2	11.2	11.2	11.2
1	67.8	8.5	8.5	8.5	8.5	8.5	33.9	17.0	8.5	8.5	8.5
2	150.8	37.1	37.1	9.3	9.3	9.3	18.6	9.3	9.3	9.3	9.3
3	471.5	60.1	60.1	60.1	30.0	15.0	30.0	15.0	15.0	15.0	15.0
4	23.7	6.0	3.0	11.9	11.9	3.0	6.0	3.0	3.0	3.0	3.0

Supplementary Table S3. Antimicrobial Selectivity Index of LfcinB and selected conjugates in relation to IC_{50} values for HL60, BT20, A549, and HEK293. For MIC and IC_{50} data, please also refer to Ptaszyńska et al., 2019. Abbreviations: S. e. - *Staphylococcus epidermidis* ATCC 12228; B. s. - *Bacillus subtilis* ATCC 6633; S. a. - *Staphylococcus aureus* ATCC 25923; P. a - *Pseudomonas aeruginosa* ATCC 27853; B. c - *Bacillus cereus* PCM 2003; C. k. - *Candida krusei* DSM 6128; C. p. - *Candida parapsilosis* DSM 5784; C. a. - *Candida albicans* ATCC 10231; C. g. - *Candida glabrata* DSM 11226; C. t. - *Candida tropicalis* CZD 519.

Malignancy Selectivity Index				
**No.**	HL60			
	HL-60	BT20	A549	HEK293
LfcinB	n/d	8.0	15.6	51.6
1	n/d	3.0	8.9	39.4
2	n/d	2.7	31.1	38.8
3	n/d	7.2	12.4	135.3
4	n/d	3.1	3.8	8.3
	BT20			
	HL-60	BT20	A549	HEK293
LfcinB	0.1	n/d	2.0	6.5
1	0.3	n/d	2.9	13.1
2	0.4	n/d	11.7	14.6
3	0.1	n/d	1.7	18.8
4	0.3	n/d	1.2	2.7
	A549			
	HL-60	BT20	A549	HEK293
LfcinB	0.1	0.5	n/d	3.3
1	0.1	0.3	n/d	4.4
2	0.0	0.1	n/d	1.3
3	0.1	0.6	n/d	10.9
4	0.3	0.8	n/d	2.2
	HEK293			
	HL-60	BT20	A549	HEK293
LfcinB	0.0	0.2	0.3	n/d
1	0.0	0.1	0.2	n/d
2	0.0	0.1	0.8	n/d
3	0.0	0.1	0.1	n/d
4	0.1	0.4	0.5	n/d

Supplementary Table S4. Normalized to EC_{50} Malignancy Selectivity Index.

**Compound**	**MW [g/mol] (founzd/calc.).**	t_R **[min]**	**Yield %**
CIP	331.4	11.3	--
LVX	361.4	11.2	--
FLC	306.3	10.7	--
FLC-COOH	365.2/364	11.8	46%
CIP-Cys	435.3/434.5	29.3	92%
LfcinB(2-11)-NH_2	1496.9/1495.8	14.3	90-95%
Nle- LfcinB-NH_2	2017.8/2017.5	15.3	
1	1841.2/1839.2	15.6	4%
2	1929.6/1929.8	16.2	30%
3	2452.2/2451.5	15.5	30%
4	1843.6/1841.8	18.3	73%

Supplementary Table S5. Physicochemical properties of peptide conjugates and its components.

Supplementary Figure S1. MS analysis of peptide and peptide conjugates and their constituents. (a) CIP-Cys; (b) LfcinB(2-11)-NH2; (c) Nle- LFcinB-NH2; (d) 1; (e) 2; (f) 3; (g) 4.

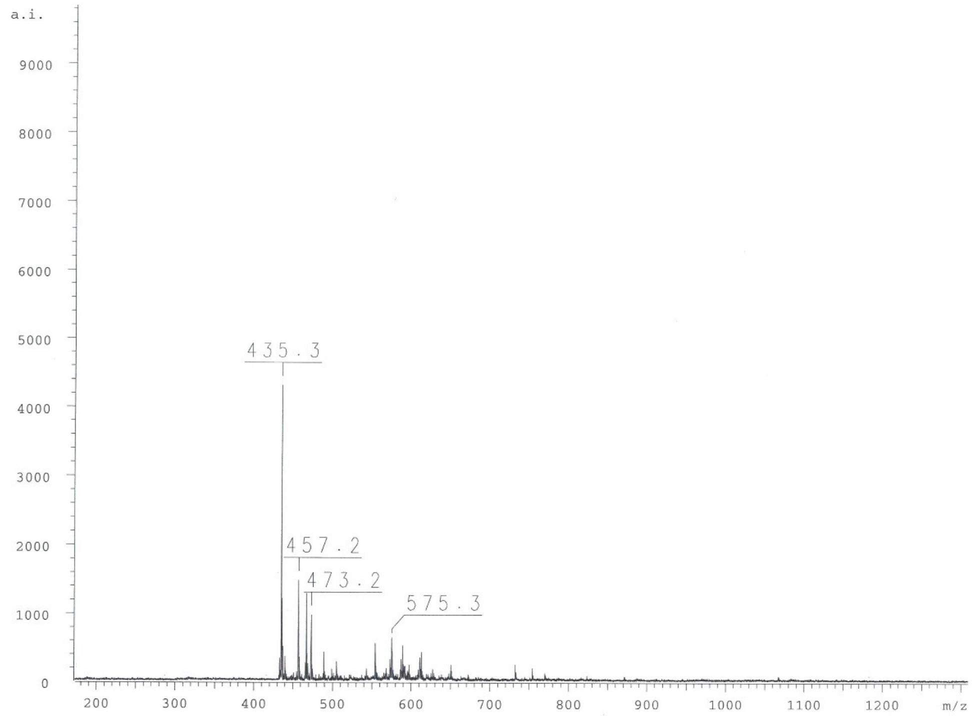

(a) CIP-Cys

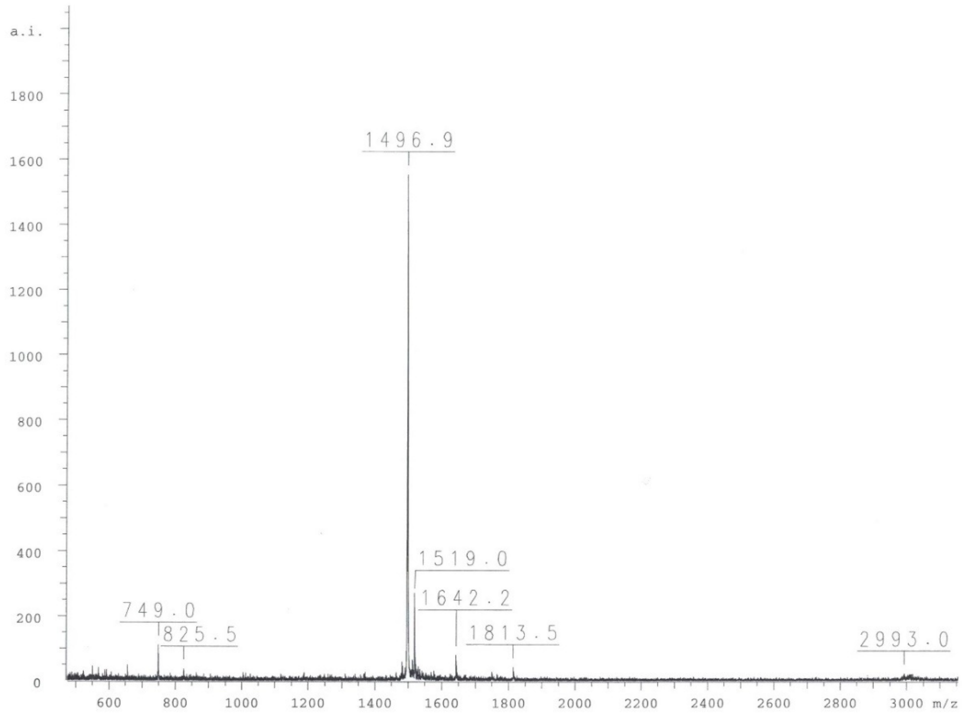

(b) LfcinB(2-11)-NH_2

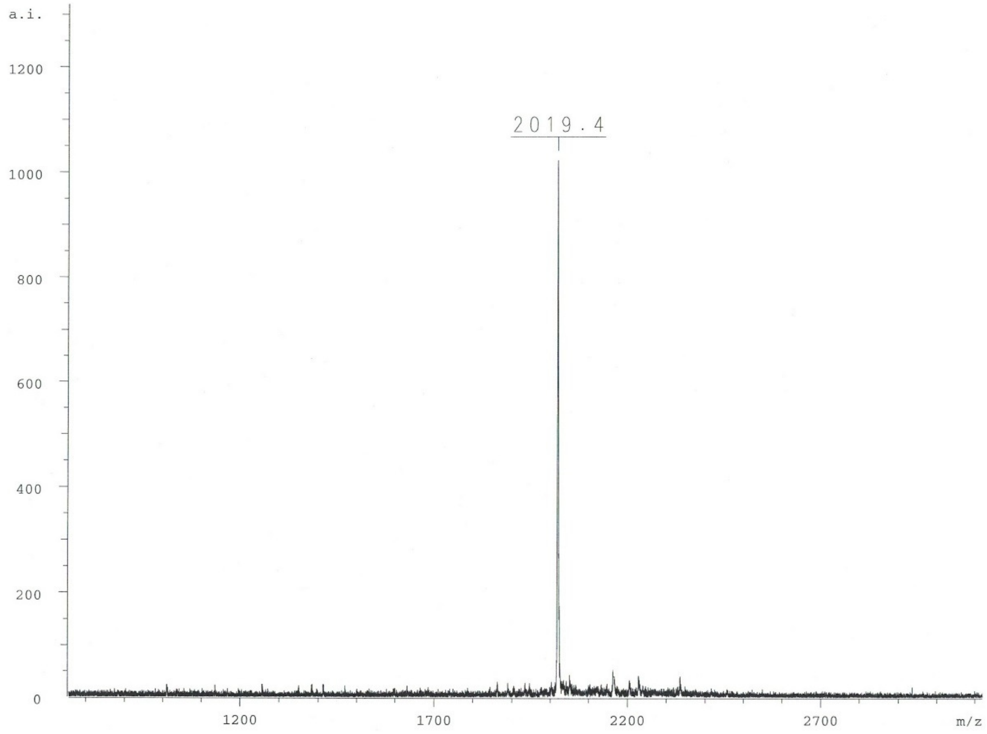

(c) Nle-LFcinB-NH2

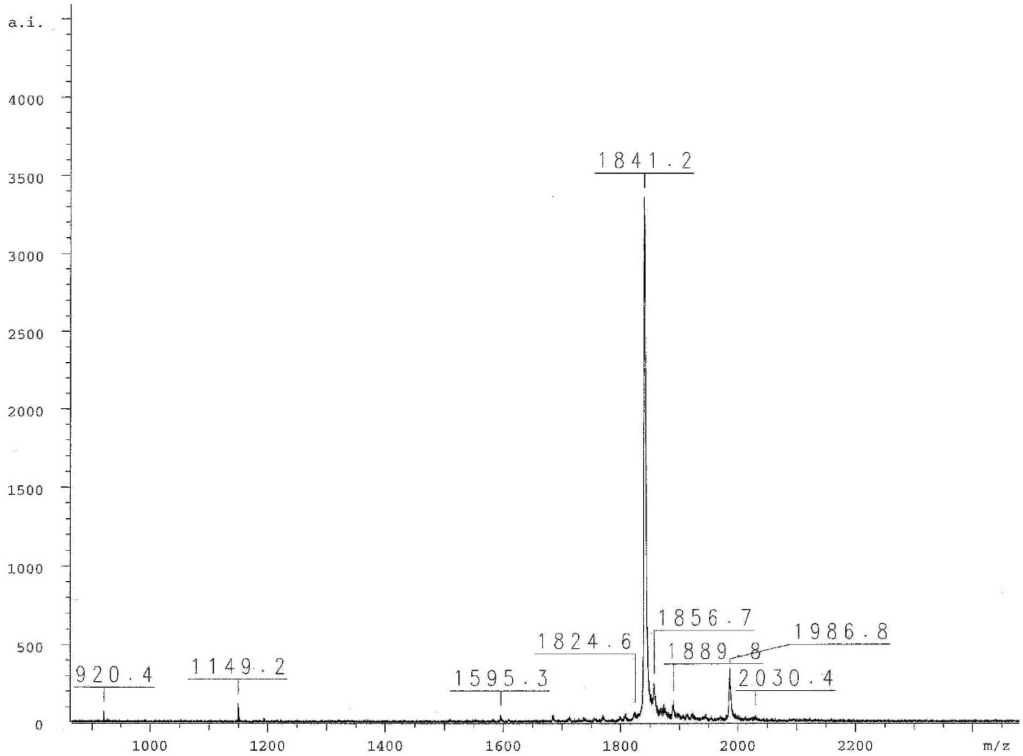

(d) 1

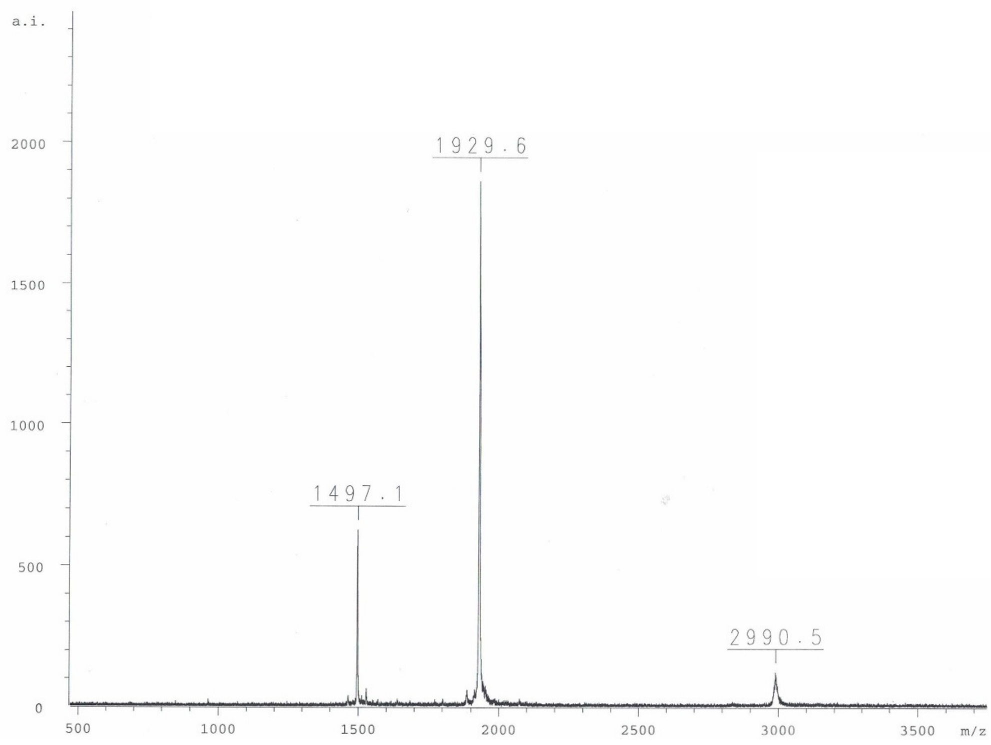

a.i.
2000
1500
1000
500
0
1929.6
1497.1
2990.5
500
1000
1500
2000
2500
3000
3500
m/z

(e) 2

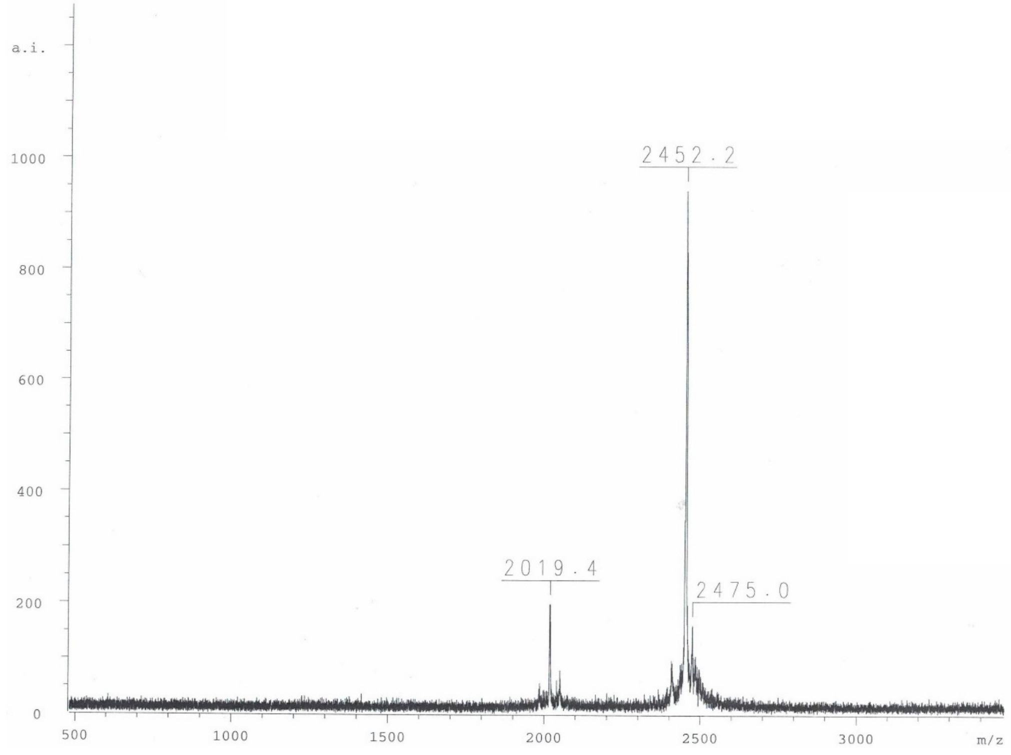

a.i.
1000
800
600
400
200
0
2452.2
2019.4
2475.0
500
1000
1500
2000
2500
3000
m/z

(f) 3

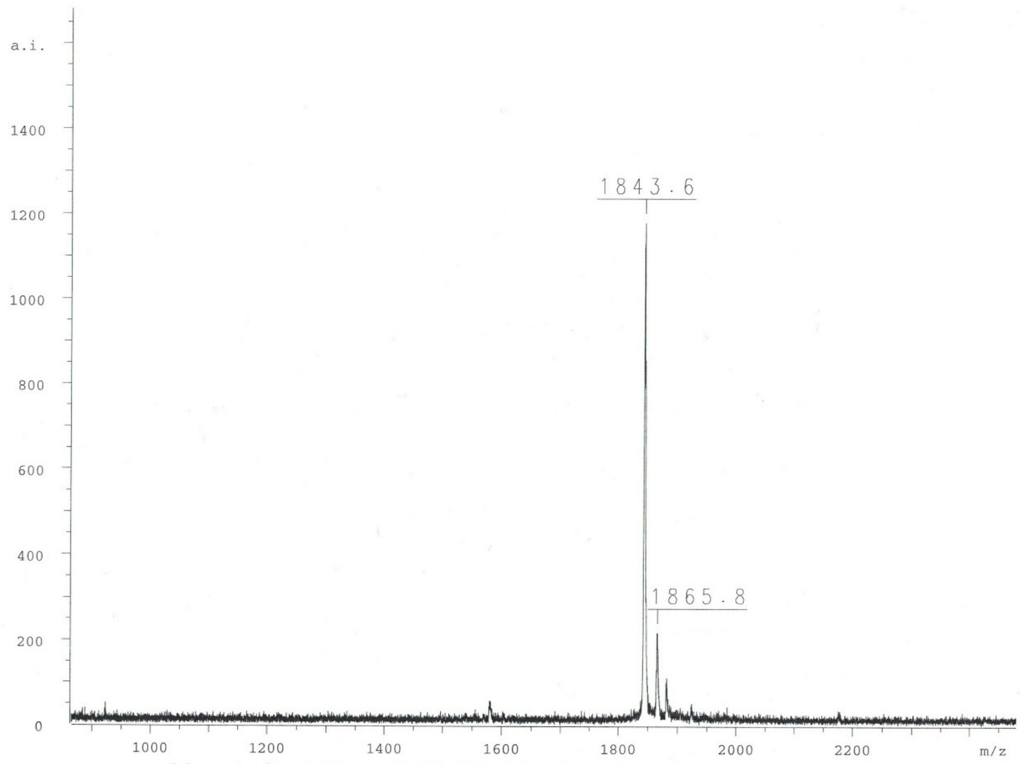
a.i.
1400
1200
1000
800
600
400
200
0
1843.6
1865.8
1000
1200
1400
1600
1800
2000
2200
m/z
(g) 4
